# Chemical Modification of Hyaluronan and Their Biomedical Applications

**DOI:** 10.3389/fchem.2022.830671

**Published:** 2022-02-11

**Authors:** Vera Hintze, Matthias Schnabelrauch, Sandra Rother

**Affiliations:** ^1^ Institute of Materials Science, Max Bergmann Center of Biomaterials, Technische Universität Dresden, Dresden, Germany; ^2^ Biomaterials Department, INNOVENT e. V., Jena, Germany; ^3^ School of Medicine, Center for Molecular Signaling (PZMS), Saarland University, Homburg, Germany

**Keywords:** hyaluronan, glycosaminoglycan, synthesis, interaction, signaling, degradation

## Abstract

Hyaluronan, the extracellular matrix glycosaminoglycan, is an important structural component of many tissues playing a critical role in a variety of biological contexts. This makes hyaluronan, which can be biotechnologically produced in large scale, an attractive starting polymer for chemical modifications. This review provides a broad overview of different synthesis strategies used for modulating the biological as well as material properties of this polysaccharide. We discuss current advances and challenges of derivatization reactions targeting the primary and secondary hydroxyl groups or carboxylic acid groups and the *N*-acetyl groups after deamidation. In addition, we give examples for approaches using hyaluronan as biomedical polymer matrix and consequences of chemical modifications on the interaction of hyaluronan with cells via receptor-mediated signaling. Collectively, hyaluronan derivatives play a significant role in biomedical research and applications indicating the great promise for future innovative therapies.

## Introduction

Hyaluronan (HA, formerly named hyaluronic acid) is an ionic, non-branched and multifunctional heteropolysaccharide. Together with other low- or medium-sulfated representatives (heparan sulfate, chondroitin sulfate, dermatan sulfate, keratan sulfate) and the high-sulfated heparin, the non-sulfated HA belongs to the family of naturally occurring glycosaminoglycan (GAG) biomacromolecules, the so called mucopolysaccharides. GAG are found throughout the body, often in mucus and joint fluids, and as components of the extracellular matrix (ECM) and tissues of vertebrates and invertebrates. GAG are localized also inside and on the surface of all cells. They are involved in various biochemical processes such as cell adhesion, growth and proliferation, cell surface binding, wound healing, or tumor metastasis (Dicker et al., 2014).

Compared to sulfated GAG, HA is a structurally uniform natural macromolecule and due to established biotechnological production processes it is readily available in higher quantities (Badri et al., 2018). Despite the progress achieved in the total synthesis of complex oligomeric carbohydrates (DeAngelis et al., 2013; Mende et al., 2016; Fittolani et al., 2021), HA represents an ideal starting material for the chemical conversion into other hardly accessible, mainly high-sulfated GAG or carbohydrate-analogous polymeric molecules mimicking their function, e.g., in the interactions with proteins like mediator molecules (e.g. cytokines).

There are two main features of HA that have contributed to its attractiveness as a biomaterial. Firstly HA can act as a passive structural molecule. Due to its macromolecular size, the marked hygroscopicity and viscoelasticity, HA is able to modulate tissue hydration, and to act as an osmotic balance. As a component of the ECM, HA can provide an extracellular space, where cells and various other ECM compounds like collagen or elastin fibers are firmly maintained. In an active way, HA is able to act as signaling molecule interacting with various receptor proteins, namely extracellular and cellular hyaloadherins (Fallacara et al., 2018). In this function HA is involved in angiogenesis, cell migration and motility, and tissue organization. It also plays a role in inflammation and stimulation of cytokine activity.

Secondly, HA can be functionalized and chemically modified to present a range of physical characteristics with wide-ranging solubility and even mechanical properties. HA is highly soluble in aqueous media, especially at low pH, and it has a high rate of turnover in human tissue. At higher concentrations HA forms physically crosslinked gels of often unsatisfactory mechanical stability.

Both, the relatively rapid degradability of HA in living tissues and its low mechanical stability are often a challenge for the broad applicability of HA in the clinic as a versatile biomaterial. Classical applications of HA include currently injections into joint spaces for the treatment of osteoarthritis or into the eye to increase the viscosity of the vitreous humor as well as wound carrier materials based on HA-esters or crosslinked HA derivatives.

The synthesis of new HA derivatives should take into account the multifunctionality of the HA molecule by selecting highly regioselective syntheses applying mild reaction conditions. Special attention should also be paid to the properties of native HA as its excellent biocompatibility, adjustable biodegradability, and mucoadhesivity.

Based on the structural peculiarities and the multifunctional character of the high molecular-weight HA molecule, recent advancements in the selective chemical functionalization of HA are presented in the following. These selectively modified derivatives are intended, on the one hand, to mimic more complex GAG or GAG derivatives that can be synthesized only in time-consuming multi-step syntheses and high personal efforts. On the other hand, significant contributions to the detailed understanding of important interaction processes between GAG and their different reaction partners like ECM proteins, intracellular proteins or specific growth factors (Grosskopf et al., 2021) are expected.

## Occurrence, Biosynthesis, and Degradation

GAG occur as the constituents of so-called proteoglycans (all GAG members except HA) forming a large heterogeneous family of macromolecules consisting of a central protein backbone to which one or more unbranched GAG chains are covalently linked (Pomin and Mulloy, 2018). In case of cell surface proteoglycans, the protein core is membrane-spanning or lipid-bound, while ECM proteoglycans are predominantly secreted. HA is widely distributed in both prokaryotic and eukaryotic cells. Adult humans contain about 15 g of HA (Fallacara et al., 2018), mostly occurring in skin, vitreous body of the eye, umbilical cord, synovial fluid of articular joints, intervertebral disks, and embryonic mesenchymal tissues. In addition, it is present in further tissues such as heart valves, lungs, tendon sheaths, bursas aorta, and prostate (Valachová and Šoltés, 2021).

**FIGURE 1:**
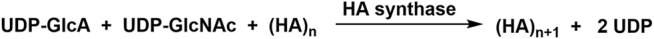
Overall reaction of the chain elongation step during HA biosynthesis.

Biosynthesis of HA occurs in eukaryotic cells at the inner surface of the plasma membrane catalyzed by a class of integral membrane proteins, the HA synthases (HAS1, HAS2, HAS3; Vigetti et al., 2014). These enzymes which contain both GlcNAc (β-1 → 4) and GlcA (β-1 → 3) transferase activities, elongate HA at its reducing end by repeatedly adding nucleotide [uridine-diphosphate (UDP)]-activated GlcA and UDP-GlcNAc to the nascent polysaccharide (Hascall and Esko, 2009). The overall reaction scheme is given in Figure 1 (Schnabelrauch et al., 2013). Finally, the synthesized HA is extruded through the plasma membrane into the extracellular space. A comparable pathway to produce HA is used by bacteria leading to HA with identical structural features (DeAngelis, 2012).

The first pharmaceutically pure HA was produced from rooster combs using the extraction and purification method by Balazs (Balazs, 1979). Currently, industrial production of HA mainly occurs by microbial fermentation (Boeriu et al., 2013) using at first Streptococci strains A and C, and more recently, various commercially available strains like *Streptococcus equi*, or *Streptococcus zooepidemicus* and even genetically modified bacterial strains (Moradali and Rehm, 2020; Manfrão-Netto et al., 2021). Even though high amounts of HA occur within the glycocalyx of all cells and in the ECM, the plasma HA levels are usually low due to the rapid clearance of HA in the kidney and liver (Fraser et al., 1997). About one-third of the total HA amounts in the human body are subject to daily turnover. HA degradation is mainly achieved by the activity of hyaluronidases, which catalyze the hydrolysis of disaccharides at hexosaminidic (β-1 → 4) linkages. In most tissues, among the six identified hyaluronidases, HYAL1 and HYAL2 are the dominating ones. Cell surface bound HYAL2 degrades high-molecular-weight HA into HA fragments of around 50 disaccharide units (about 20 kDa), which stimulate angiogenic and inflammatory signaling pathways (Stern, 2004). After internalization of these fragments into endosomes and transport to lysosomes, HYAL1 cleaves the HA fragments into small oligosaccharides before further degradation by exoglycosidases (Stern et al., 2006).

## General Structure and Properties

HA is a negatively charged, unbranched and multi-functional polysaccharide formed by repeating disaccharide units of D-N-acetylglucosamine (GlcNAc) and D-glucuronic acid (GlcA) linked by alternating (β-1→3) and (β-1→4) glycosidic bonds. In contrast to the other members of the GAG family, HA is not sulfated. At physiological pH (around 7.4), most carboxyl groups are deprotonated and, therefore, HA (pK ≈ 3.2) is negatively charged. In solution and at physiological pH value, the negative charge of HA is balanced with different cations (e.g., Na^+^, K^+^, Ca^2+^, and Mg^2+^). Furthermore, HA can chelate and neutralize iron and copper ions, which are required for the Fenton reaction and are responsible to form the most deleterious reactive oxygen species (ROS) (Gligorovski et al., 2015). In the absence of iron and copper ions, the formation of these hydroxyl radicals is supressed. Moreover, HA can also neutralize ROS around leukocytes and protect neighboring cells (Litwiniuk et al., 2016).

**FIGURE 2:**
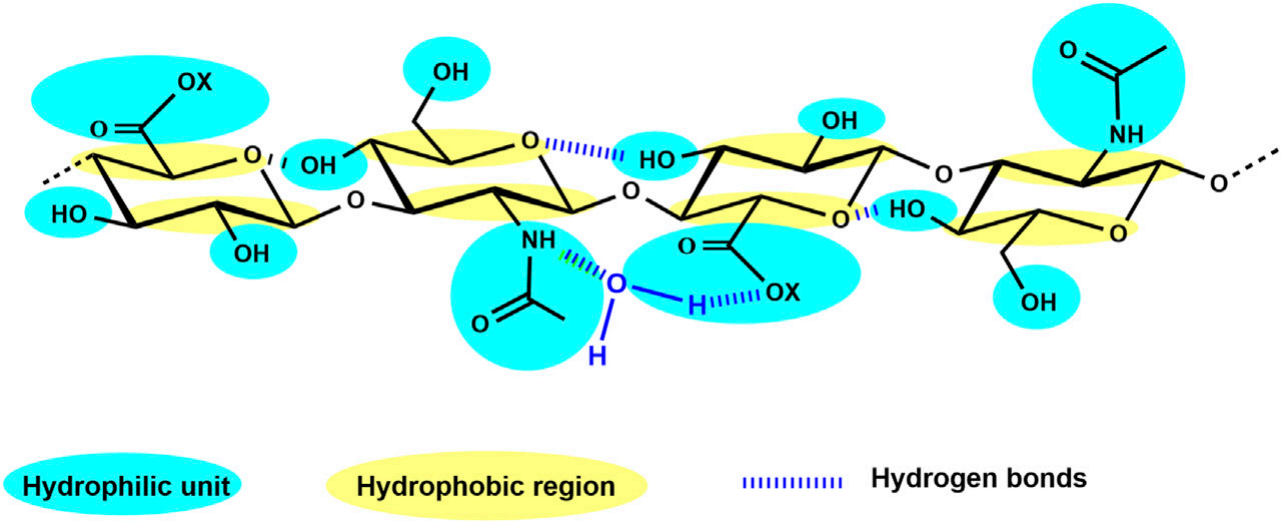
HA tetrasaccharide unit schematically showing the hydrophilic moieties (in blue) and the hydrophobic regions (in yellow), while hydrogen bonds are represented by dark blue dashed lines (adapted from Fallacara et al., 2018, 10, 701).

Concluded from experimental findings and supported by various spectroscopic analyses and first data from energy calculations as well as computer simulations at the beginning of the 1990s, a twofold helix for HA was hypothesized to be present in aqueous solution (Heatley and Scott, 1988; Scott et al., 1991). The formed double helix is probably driven by interactions between large hydrophobic patches on alternate sides of the tape-like polymer and additionally by hydrogen bonding, forming stable aggregates at biological temperatures in water (Scott, 1992). This interplay of hydrophilic nature, hydrophobic portions, and a strong network of hydrogen bonds (Figure 2) results in the generation of an unstable β-sheet tertiary structure (Amorim et al., 2021). Although rather simply structured, compared to the other GAG, HA shows remarkable physico-chemical and biological properties (Bohaumilitzky et al., 2017). It is a very hygroscopic macromolecule able to tightly bind 15 water molecules per disaccharide repeating unit (Jouon et al., 1995) and has a great ability to retain water. Furthermore, HA shows a very high and shear-dependent viscoelasticity, resulting in the role of HA as an extracellular lubricant (Laurent et al., 1996). These remarkable hydrodynamic properties are essential for the biological function of HA to maintain tissue hydration, tension, and integrity.

The molecular weight of HA is a fundamental molecular parameter that has to be determined with high accuracy. Especially, the biological properties of HA and its synthesized derivatives largely depend on its molecular weight. Gel permeation chromatography (GPC), a special form of size exclusion chromatograpy (SEC), equipped with a multiangle laser light scattering (MALLS) detection system is the most commonly used analytical technique for determining the average molecular weight and molecular weight distribution (Botha et al., 2018; Schnabelrauch et al., 2021).

Depending on the biological source, HA may possess a very high molecular weight of about 10^7^ Dalton, e.g. as component in articular cartilage or of the synovial fluid in healthy joints (Fraser et al., 1997). In the physiological situation, there is a rapid HA turnover resulting in the constant presence of distinct forms of HA of various molecular weight (Bohaumilitzky et al., 2017). High-molecular weight HA (HMW-HA with molecular weights ranging from 1,000–5,000 kDa) possesses anti-inflammatory, anti-proliferative, anti-angiogenic, and immunosuppressive properties (Dovedytis et al., 2020). Besides providing water homeostasis, it modulates cell proliferation and differentiation, and participates in several other important biological processes, e.g. tissue regeneration, wound healing, epithelial integrity, embryogenesis, plasma protein distribution, and matrix structuring (Valachová and Šoltés, 2021).

## Chemical Modification of HA

### General Remarks

**FIGURE 3:**
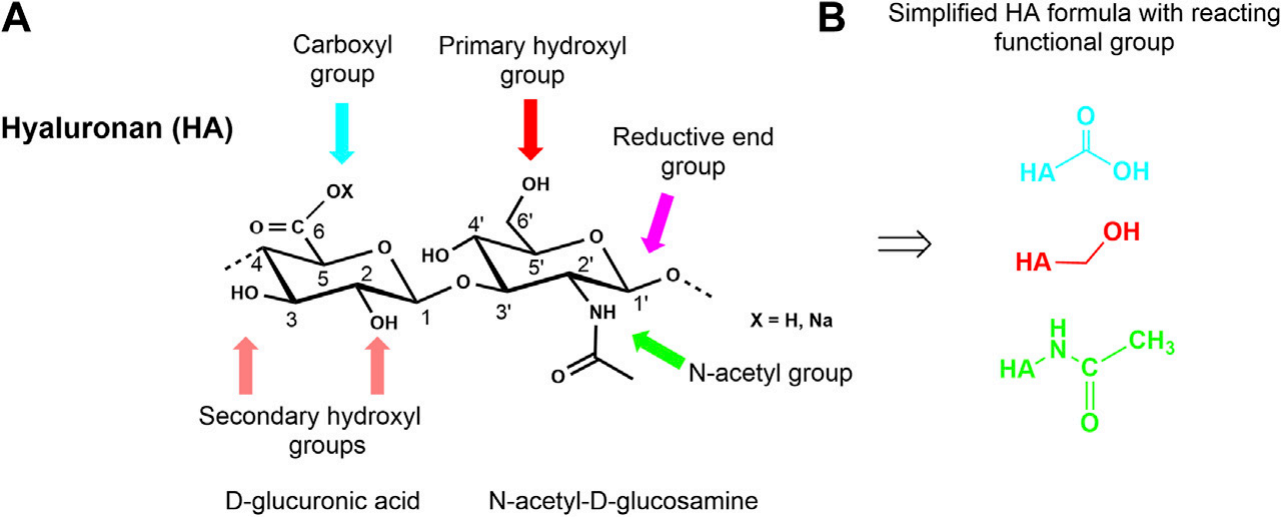
Chemical structure of a HA disaccharide repeating unit showing the reactive functional groups **(A)** and presentation of simplified HA formulas with reaction centers involved in the different modifications **(B)**.

HA, like the other GAG, is a multifunctional macromolecule bearing primary and secondary hydroxyl, carboxyl, and N-acetyl groups within each anhydrodisaccharide repeating unit and a reducing sugar end group at the HA chain terminus (Figure 3A). The carboxylate group is responsible for the anionic character of the polysaccharide and makes HA soluble in aqueous solutions. However, the presence of the ionic functionality in combination with an intra- and intermolecular hydrogen-bonding network makes HA insoluble in conventional organic solvents. Nevertheless, it is possible to transform the sodium salt or the acidic form of HA into tertiary or quaternary ammonium salts of HA rendering such salts soluble or at least highly swellable in aprotic solvents like N,N-dimethyl formamide (DMF) or dimethyl sulfoxide (DMSO). These solvents usually allow esterification reactions under nearly homogeneous reaction conditions (Moeller et al., 2012). Some other solvent systems like ionic liquids (Zakrzewska et al., 2010) have already been used to modify other non-GAG polysaccharides or have been used for the extraction of GAG- and ECM-containing proteins as supercritical carbon dioxide (Wang et al., 2017). Another aspect that must be considered when chemically manipulating HA and other GAG is their sensitivity to oxidative agents as well as to thermal stress.

Overall, the multi-functionality of HA (Figure 3B) combined with its limited solubility in organic solvents and its sensitivity to enzymatic and thermal degradation make the discovery of selective chemical reactions a challenge for the synthetic chemist.

In principle, HA can be modified by two different methods, crosslinking, and conjugation. Both methods are based on the same chemical reaction and differ in the fact that conjugation is characterized by grafting a single bond onto a HA chain whereas crosslinking means that different HA chains are linked together by two or more bonds (Schanté et al., 2011). Crosslinking is a well-known method to improve the mechanical, swelling, and rheological properties of HA. In this way, the degradation time of HA materials is slowed down and their residence time at the respective application site is enhanced. Covalent crosslinking provides the opportunity to provide hydrogels or cryogels, sponges, and other solid forms as scaffolds and drug carriers whilst maintaining biological functionality over an adjustable period of time (Williams, 2014).

Conjugation involves a variety of chemical modifications, such as the introduction of special functional units including ether, ester or amide groups, the attachment of bioactive as well as prodrug moieties, and last but not least the incorporation of marker molecules like specific dyes. Often an approach to chemically modify HA, contains both conjugation and cross-linking processes.

In recent years, the introduction of additional anionic groups, especially sulfate half-ester groups in HA has received much attention. Such highly-sulfated HA derivatives are able, similar to heparin and heparan sulfate, but in contrast to unsulfated HA, to interact with biological mediator molecules (e.g., growth factors) and positively influence biochemical processes, e.g. wound healing (for a detailed discussion, please see one of the next chapters on interaction of chemically modified HA with biological mediator proteins). Another example is the grafting of long hydrophobic chains onto HA. Amphiphilic HA polymers of this type can form strong physical interactions which are broken when submitted to high shear forces (Huin-Amargier et al., 2005). Compared to native HA, the shear-thinning behaviour of these HA derivatives is much more marked. An important property of native HA is its mucoadhesivity. Maintaining or improving mucoadhesive properties of HA derivatives is initially important for the development of effective drug release systems. Through thiolation of HA not only mucoadhesiveness can be improved, but also properties as swelling capacity, stability, enzyme inhibition properties, and biocompatibility can be enhanced (Griesser et al., 2018).

### Controlled Degradation to Provide Suitable Starting Hyaluronans for Chemical Modification

The high solution viscosity of native HA in aqueous or organic solvents necessitates homogeneous reaction conditions as complete solubility and good miscibility. Therefore, the controlled degradation of HMW-HA to starting materials with molecular weights of 500–20 kDa prior to chemical modification reactions might often be an advantage (Schnabelrauch et al., 2021). Retaining the original macromolecular structure in form of a degraded material free of undesired side products especially without non-saturated or oxidized moieties is essential. Further, there is another critical point concerning the degradation as well as the medical use of HA products. It is well accepted that HA can modulate many biological processes including cell adhesion, cell migration, morphogenesis, tumorigenesis, cell survival, apoptosis, and inflammation and that these biological effects can (and often do) differ depending on the HA molecular weight (Cyphert et al., 2015; Fallacara et al., 2018). The mentioned complications to ensure largely homogeneous chemical HA modifications and the increasing application of HA as a component of artificial matrices and in bioengineering for tissue scaffolding, makes it necessary to develop procedures leading to HA starting materials with a controlled range of molecular weight and a narrow dispersity.

In the human body the degradation of HA is either specifically mediated by a group of endoglycosidases, the so-called hyaluronidases or non-specifically by oxidative damage due to initiation of reactive oxidative species (ROS) (Stern et al., 2007; Fallacara et al., 2018). In recent years, several enzymatic processes have been established to provide purified and structurally characterized HA oligosaccharides in up to milligram-/gram-scale quantities (Tawada et al., 2002; Blundell at al. 2006; Koehling et al., 2016). Degradation processes for HMW-HA have also been developed under largely controlled laboratory conditions by means of oxidative processes (Duan and Kasper, 2011). As oxidation agents mainly ozone (Yue 2012; Rother et al., 2015) or chlorine dioxide (Wyrwa et al., 2012) have been used. In recent years, intensive studies have been performed about acidic degradation of HA (Smejkalová et al., 2012a; Cozíková et al., 2017). Degraded HA with very narrow dispersity (Mw/Mn < 1.23) covering a broad range of differently sized products (420–3 kDa) could thus be obtained prior fractionation on a preparative scale. Based on earlier work on the thermal stability (Bothner et al., 1988; Lowry and Beavers, 1994), a simple thermal degradation process for HA was recently established (Kunze et al., 2010). Starting with a HA of weight-average molecular weight (M_w_) of 1,000 kDa the final M_w_-value of low molecular weight HA (LMW-HA) can be adjusted by choosing the processing time between 30 and 240 min resulting in M_w_ from 500 to 6 kDa (Schnabelrauch et al., 2021). Furthermore, automated solid-phase synthesis was used to produce HA oligosaccharides from monosaccharide building blocks, however, only in low amounts (Walvoort et al., 2012).

### Functionalization of the HA Hydroxyl Group

#### Alkylation Reactions

**FIGURE 4:**
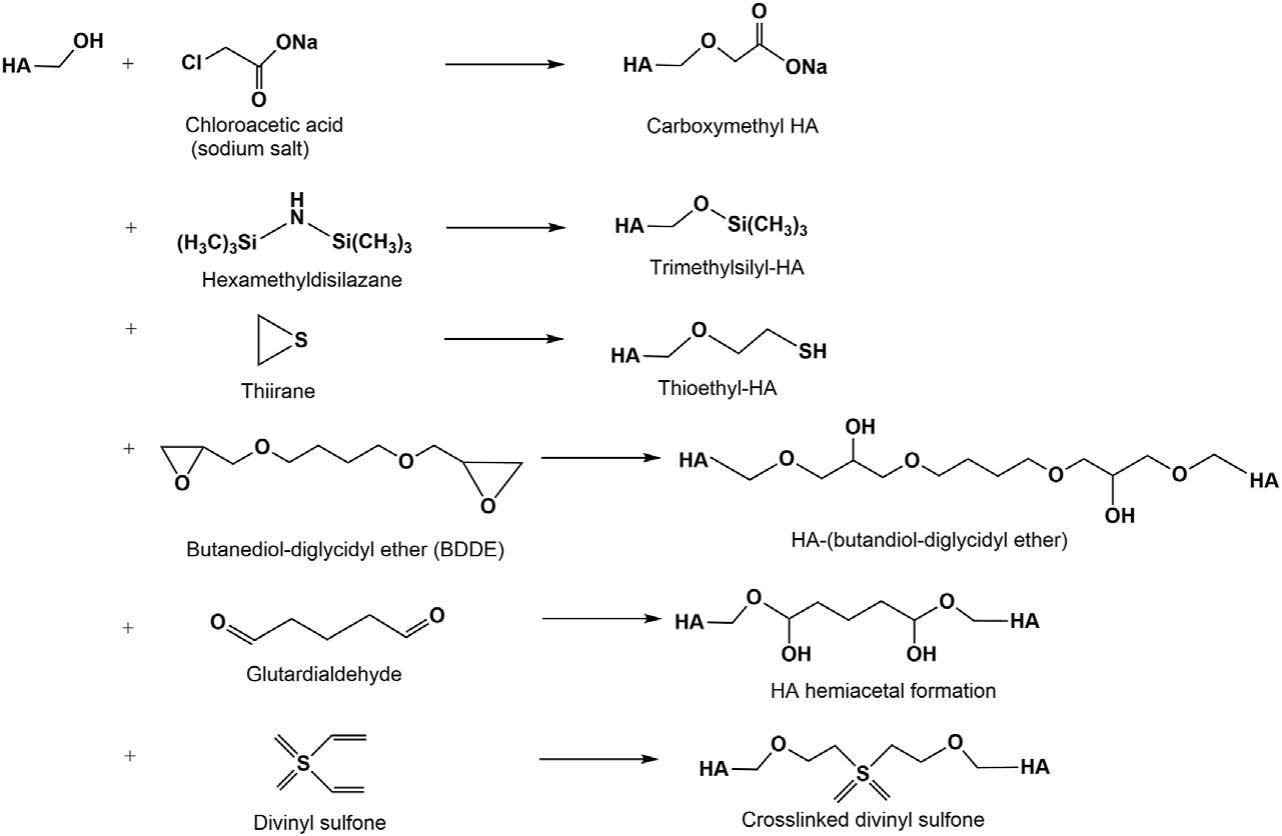
Alkylation and bis-alkylation (crosslinking) of hydroxyl groups of HA.

The modification of hydroxyl group containing biopolymers with mono- and dialkylating agents to form ethers is a classical reaction for many polysaccharides (Heinze et al., 2018). In the HA chemistry this reaction is especially used for crosslinking reactions resulting in hydrogels or scaffold materials (Figure 4). The synthesis of HA ethers is limited to a few examples due to harsh reaction conditions (pH 13-14) often necessary for the etherification reaction. Water soluble alkyl derivatives of HA have been prepared by reaction of HA with 2-alkyloxymethyloxirane in DMSO under slightly alkaline conditions (Mlbochová et al., 2007). Another approach to HA ethers described the tosylation of the primary hydroxyl group of HA followed by exchange of the more reactive tosylate functionality by hydrophobic hexyl- and pentadecyl ether groups, resp. (Lapcik. et al., 2010). Carboxymethylated HA (CM-HA) can be synthesized in analogy to other carboxymethylated polysaccharides (Yang et al., 2010) by reacting HA with monochloroacetic acid under alkaline conditions (Moeller et al., 2012). Degrees of carboxymethylation (DS_CM_) for HMW- and LMW-HA ranging between 0.2 and 0.5 are obtained after a single carboxymethylation step. A repeated carboxymethylation gave a slight increase of the DS_CM_-values to about 0.6–0.8. As shown by ^13^C-NMR studies, carboxymethylation mainly took place at the primary OH-group of HA. Molecular weight determinations employed by GPC of the products confirmed a remarkable decrease after both alkylation steps. A thioethyl ether derivative was synthesized by reaction of ethylene sulfide (thiiran) to the hydroxyl groups of HA under alkaline conditions followed by addition of dithiothreitol (Serban et al., 2008). This derivative shows a radical scavenger activity.

Silylation of polysaccharides has been recognized as an effective method to prepare organic soluble derivatives and to improve their hydrophobicity (Heinze and Liebert, 2001). Silylation of HA has been reported to occur in a homogeneous salt complex of HA with cetyltrimethylammonium bromide (Scott, 1962). The silylation was performed in this salt complex homogenously using hexamethyldisilazane (HMDS) and DMSO as solvents (Zhang and James, 2005a) resulting in silylated HA materials characterized by high degree of substitution (DS) values (above 2.5), relatively low molecular weight loss, and solubility in non-polar organic solvents. Furthermore, the silylated cetyltrimethylammonium HA can be used as intermediate in an exchange reaction with aliphatic acid chloride (from hexanoyl to stearoyl acid chloride) to prepare HA esters (Zhang and James, 2005b). Trimethylsilyl chloride is formed as leaving group. The HA esters are soluble in non-polar solvents like xylenes.

Several difunctional agents have been used to crosslink hydroxyl groups of HA via ether linkages (Figure 4) forming hydrogels (Schanté et al., 2011; Collins and Birkinshaw, 2013; Khunmanee et al., 2017). Among diepoxy compounds used for the crosslinking of HA are butanediol-diglycidyl ether (Xue et al., 2020), ethylene glycol-diglycidyl ether (Oelschlaeger et al., 2016), polyethylene glycol diglycidyl ether (Calles et al., 2013), polyglycerol polyglycidyl ether (Yui et al., 1992) and 1,2,7,8 diepoxyoctane (Zhao, 2006). At present, BDDE represents the most promising diepoxide due to its easy availability and the capability to degrade into non-cytotoxic fragments (De Boulle et al., 2013). Numerous studies showed that crosslinking can be performed by hemiacetal bonds using glutardialdehyde in an acetone-water medium catalyzed at acidic conditions (Collins and Birkinshaw, 2007). However, under the same conditions, the hemiacetals can be hydrolyzed recovering the starting materials (Schanté et al., 2011). HA crosslinking is also performed with divinyl sulfone in alkaline solution (Collins and Birkinshaw, 2007; Shimojo et al., 2015).

#### Esterification Reactions

The esterification of the hydroxyl groups of HA is often a problem due to the incomplete solubility of HA in solvents suitable for acylation reactions and the lower reactivity of the secondary hydroxyl groups. Often more drastic conditions with regard to increased reaction temperature and time are needed, and in many cases only the primary hydroxyl groups are involved into the reaction. This was documented in earlier experiments on the esterification of HA with fatty acid chlorides in DMF/pyridine (Kawaguchi et al., 1992; Kawaguchi et al., 1995). In a later study HA was esterified with hexanoic anhydride under homogeneous reaction conditions at room temperature for 2 h (Smejkalová et al., 2012b). For this reason, the sodium salt of HA was previously converted into the acid form to perform the acylation in a completely dissolved state. Under the same reaction conditions, the sodium salt was esterified in a DMSO/water solution. The formed HA esters were elucidated by means of nuclear magnetic resonance (NMR) spectroscopy, and mass spectrometry. It was found that acylation in DMSO regioselectively took place at the primary hydroxyl group of the C-6 position whereas acylation in a DMSO/water solvent was carried out both at the primary C-6 position, but also at the secondary hydroxyl group of the C-4 position of the N-acetyl-D-glucosamine unit.

The anhydrides of haloacetic acids have also been used to prepare bromo- and iodoacetates of HA which are versatile precursors for the formation of HA-based hydrogels (Serban and Prestwich, 2008).

In further studies, often highly reactive acylation components like imidazole-conjugated acids (Picotti et al., 2013) or mixed anhydrides (Huerta-Angeles et al., 2014) have been used as reactive intermediates. In a similar manner, it was also possible to graft acid-activated synthetic oligo-esters derived from polylactides and poly (3-hydroxyalkanoates), resp., to the primary hydroxyl group of HA (Pravata et al., 2008; Huerta-Angeles et al., 2017). In a simple approach using an octenyl succinate half-ester as reactive acylating reagent, an amphiphilic HA could be synthesized in aqueous media (Eenschooten et al., 2010).

Unsaturated HA esters serve as promising building blocks for the generation of novel supramolecular hydrogel networks with potential applications in regenerative, cell-based research and therapies (Highley et al., 2016; D’Amora et al., 2019; Kotla et al., 2021).

**FIGURE 5:**
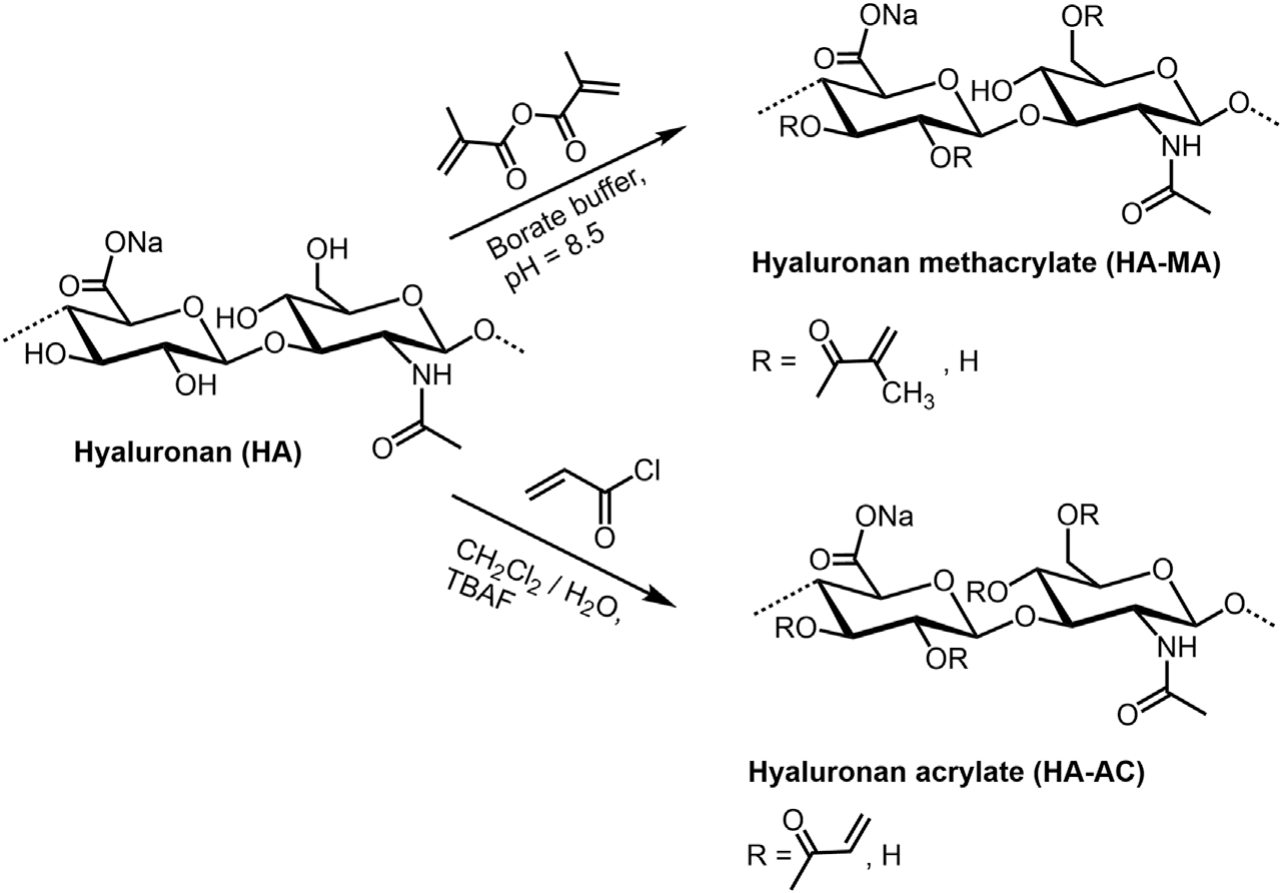
(Meth)acrylation of HA.

HA (meth)acrylates (Figure 5) are versatile macromers to generate functional coatings, and photopolymerizable scaffolds (Schnabelrauch et al., 2021). They can be prepared in a classical way by reaction of HA with methacrylic anhydride under pH values of 8–11 and ice cooling (Smeds et al., 2001; Burdick et al., 2005; Seidlits and al., 2010). The corresponding HA acrylate could be synthesized with acryolyl chloride both as mentioned above in a dichloromethane/water mixture (Qin et al., 2014), and with addition of tetra-n-butylammonium fluoride as phase-transfer catalyst (Becher et al., 2013). In analogy to non-derivatized HA, the sulfated derivatives with different degree of sulfation (DS: 0.9-1.4) can be (meth)acrylated in the same manner (Rother et al., 2017a). Further, HA esters of unsaturated carboxylic acids as pentenoates (Mergy et al., 2012) and maleates (Vasi et al., 2014) are available by treating HA with the corresponding acid anhydrides (see for example Smejkalova et al., 2014). A series of HA vinyl esters, able to be microstructured by two-photon lithography, have been synthesized by lipase-catalyzed transesterification (Qin et al., 2014).

#### Sulfate and Phosphate Esters

The biological properties of sulfated GAG are influenced to a considerable extent by the degree of sulfation and the sulfate group distribution within the disaccharide repeating units. In particular, the higher sulfated GAG can only be isolated to a limited extent with structural uniformity from biological sources and they are accessible by total synthesis only at extremely high time expenditure. For this reason, the stepwise and regioselective sulfation of HA is a time- and cost-efficient approach mimicking important biological features of high-sulfated GAG (Scharnweber et al., 2015; Townley and Bülow, 2018).

Experiments on the sulfation of HA were already carried out in the 1950s (Balazs et al., 1951). In these early experiments, sulfuric acid or chlorosulfonic acid were used as sulfating agents (Bedini et al., 2017; Caputo et al., 2019). These relatively drastic reaction conditions did not result in a complete sulfation of all free OH-groups but caused a partial degradation of the polymer chain. Therefore, in more recent attempts, complexes of SO_3_ with organic amines, especially triethylamine (Satoh et al., 2004), trimethylamine (Nagira et al., 2007), and pyridine (Magnani et al., 1996) or amides like DMF (Crescenzi et al., 2002a) were used. The SO_3_-complexes are relatively mild reagents causing less polymer degradation. Due to their different reactivities in aprotic solvents, the sulfation of HA and other GAG is adjustable to a range between 0 and 4. Preferentially, the sulfation is performed in aprotic solvents like DMF after transformation of HA in its tributyl ammonium salt (Magnani et al., 1998, Figure 6). Studies on regioselectivity of this reaction proved that the primary hydroxyl group reacts preferably. As shown by ^13^C-NMR, a sulfation of secondary hydroxyl groups was detected in HA sulfates only at a degree of sulfation (DS_S_) above 1.0 (Hintze et al., 2009). In addition to regioselective sulfation reactions, there exist two other pathways for directing sulfate groups into specific positions of HA hydroxyl moieties, namely 1) de-sulfation reactions and 2) the use of protecting groups (Bedini et al., 2016; Bedini et al., 2017). Following the former route, desulfation of a high-sulfated HA (DS_S_ = 3.1) by means of silylating agents like N-methyl-N-(trimethylsilyl)-trifluoroacetamide (MSTFA) or N,O-bis(trimethylsilyl) acetamide (BTSA) resulted in HA sulfates with DS_S_ values of 1.5 and 1.6, resp., bearing the sulfate groups mainly at the secondary hydroxyl groups as shown by ^13^C-NMR (Becher et al., 2010). In this context, it was found that the benzoyl ester group is suitable to protect the primary hydroxyl group of HA and subsequent sulfation leads to a sulfated benzoylated HA (Becher et al., 2010). Cleavage of the benzoate group under mild alkaline conditions resulted in a HA sulfate with DS_S_ values between 2.0 and 3.0. ^13^C-NMR confirmed a complete sulfation of the secondary hydroxyl groups and a free primary hydroxyl group. A subsequent esterification of the differently sulfated HA, for example, with (meth)acrylate groups result in mixed HA derivatives bearing both sulfate and (meth)acrylate units in different positions of the disaccharide repeating unit (Rother et al., 2017a; Becher et al, 2012, Figure 7). The introduction of a phosphate group into HA could be attractive for their application to biochemical systems via mediation of GAG-protein interactions. Although the phosphorylation of polysaccharides is reported in several reviews (Xu et al., 2019; Zhou and Huang, 2021), only few studies describe the phosphorylation of HA and give detailed characterization data of synthesized HA phosphates (Dulong et al., 2004; Leone et al., 2012; Bojarski et al., 2019). Phosphorylation is associated with several challenges that have not yet been satisfactorily solved. For example, the reactivity of conventional phosphorylating agents is significantly lower compared to sulfating agents, which requires harsher reaction conditions. At the same time, the tendency to crosslinking is much more pronounced with phosphorylation than with sulfation and hinders homogeneous reaction control. Chemical phosphorylation of HA and other GAG remains a challenge for the preparative polysaccharide chemistry.

**FIGURE 6:**
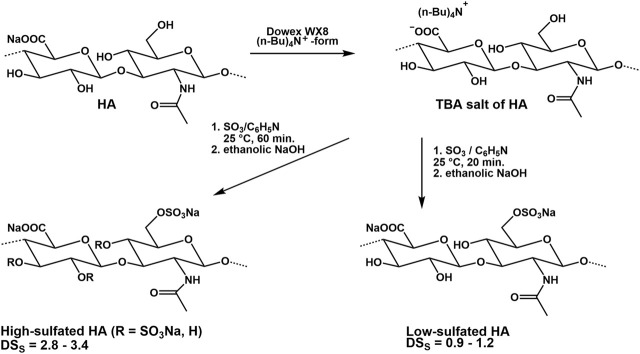
Synthesis of high- and low-sulfated HA.

**FIGURE 7:**
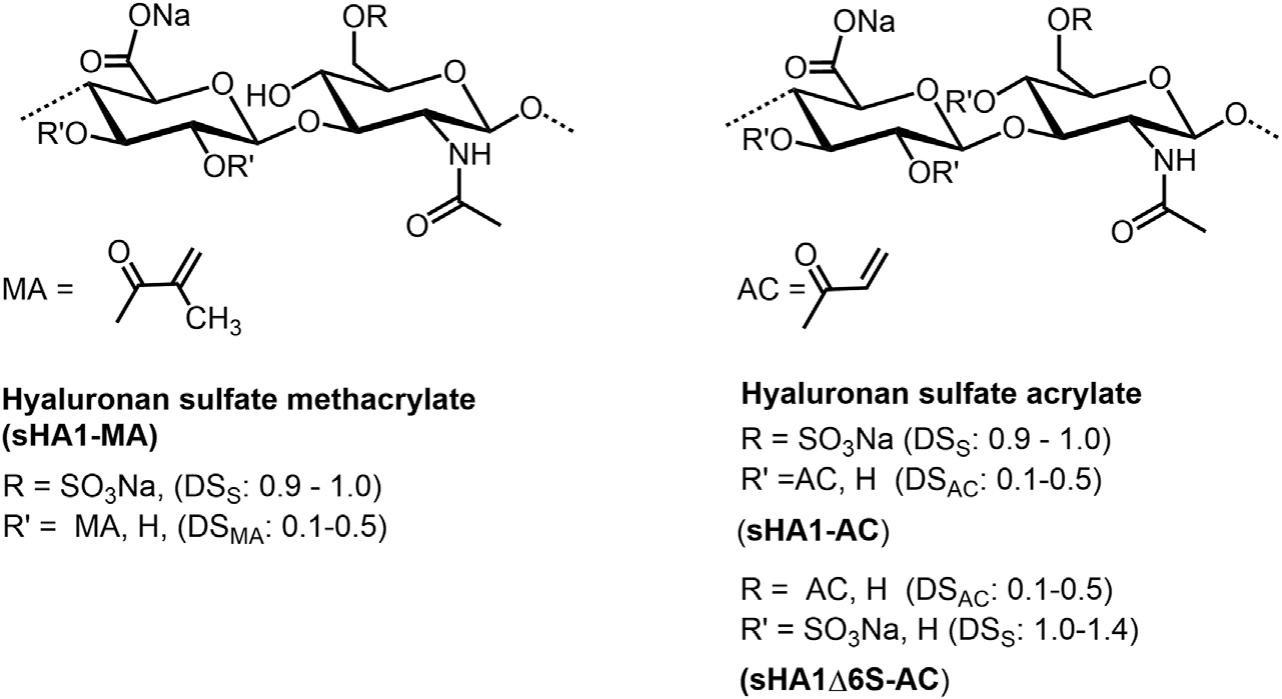
Sulfated methacrylates, and sulfated acrylates of HA.

#### Oxidation

**FIGURE 8:**
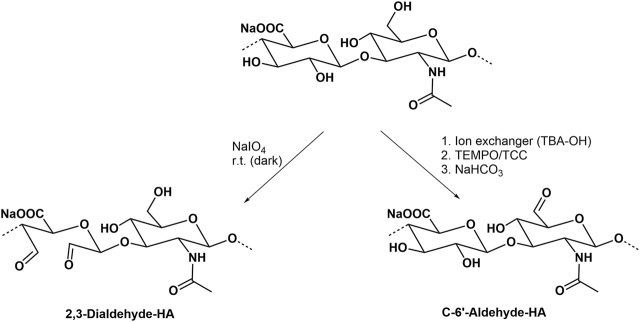
Selective oxidation of hydroxyl groups of HA.

Common oxidation reactions of polysaccharides have recently been reviewed (Palhares et al., 2021). The periodate oxidation, also called Malaprade oxidation acting on *cis*-diols which changes the polysaccharide backbone, and leads to the cleavage of the sugar ring forming corresponding carbonyl moieties (Kristiansen et al., 2010, Figure 8). Another typical regioselective oxidation of the primary hydroxyl group is mediated by the 2,2,6,6-tetramethyl-piperidinyl-1-oxy radical (Crescenzi et al., 2001; Pierre et al., 2017). The secondary hydroxyl groups and hence the sugar ring form are left unaffected. The actual oxidant in this reaction is the nitroxyl radical in form of a nitrosonium cation, which is continuously regenerated by another oxidant present in the reaction mixture. The oxidation can thus proceed to a high yield with only a catalytic amount of TEMPO (Jiang et al., 2000). In an improved version, trichloroisocyanuric acid (TCC) is used as a secondary oxidation reagent (Figure 8), both to activate and regenerate TEMPO (Shan et al., 2021). Oxidized HA derivatives resulting from both types of oxidation can serve as precursors for the formation of drug releasing hydrogels (Zhang et al., 2011) or the conjugation of proteins (Mero and Campisi, 2014).

Besides TEMPO, also periodinane (also called Dess-Martin periodinane) can be used as a relatively mild oxidation agent which similar to TEMPO is able to transform primary hydroxyl groups from HA to aldehyde functions (Durana et al., 2006; Šedová et al., 2013). Aldehyde-containing HA derivatives have recently shown a high potential to prepare conjugates with different amines in drug delivery (Buffa et al., 2018).

### Functionalization of the HA Carboxyl Group

#### Esterification

Strategies for the modification of the carboxyl group of HA also involve esterification and amidation reactions. Well-known biomaterials synthesized in this context are the family of HYAFF materials, especially the benzyl (HYAFF 11) and the ethyl ester (HYAFF 7) of HA (Benedetti et al., 1990; Benedetti et al., 1993). The HA esters have been prepared from the corresponding alcohols after dissolution of the quaternary HA in an aprotic solvent (Campoccia et al., 1998).

The reaction of epoxides with the carboxyl group of HA is in particular reported for the reaction with glycidyl methacrylate to generate methacrylate ester under aqueous conditions with catalytic amounts of triethylamine (Baier Leach et al., 2003; Moeller et al., 2007). As shown by ^13^C-NMR studies (Bencherif et al., 2008) at pH 7.4 two reactions occur simultaneously, namely an irreversible ring-opening conjugation through the HA carboxylic group toward the highest substituted carbon of the epoxide and a reversible transesterification through the primary hydroxyl group of HA.

#### Amidation

Amidation of the carboxyl group of HA with amines, hydrazins, and hydrazides is one of the most widely used method in the synthesis of new HA amides (Schanté et al., 2011), the design of novel delivery systems (Oh et al., 2010), and also in HA carboxy-based network formation (Burdick and Prestwich, 2011). To initiate amidation, normally the carboxyl group of HA has to be activated. Typical activation agents used are water-soluble carbodiimides like 1-ethyl-3-[3-(dimethylamino)-propyl]-carbodiimide (EDC), often used in combination with 1-hydroxybenzotriazole (HOBt) or N-hydroxysulfosuccinimide (sulfo-NHS) (Bulpitt and Aeschlimann, 1999). Further activating agents are 1,1′-carbonyldiimidazole (D’Este et al., 2012), 2-chloro-1-methylpyridinium iodide (Magnani et al., 2000), 2-chloro-dimethoxy-1,3,5-triazine (Bergman et al., 2007), and 4-(4,6-dimethoxy-1,3,5-triazin-2-yl)-4-methylmorpholinium chloride (D’ Este et al., 2014, Figure 9). The advantages of DMTMM conjugation, as a powerful tool to synthesize tyramine modified HA hydrogels, which are bio-orthogonally crosslinked with horseradish peroxidase (HRP) and hydrogen peroxide (H_2_O_2_) have also been demonstrated (Loebel et al., 2015).

**FIGURE 9:**
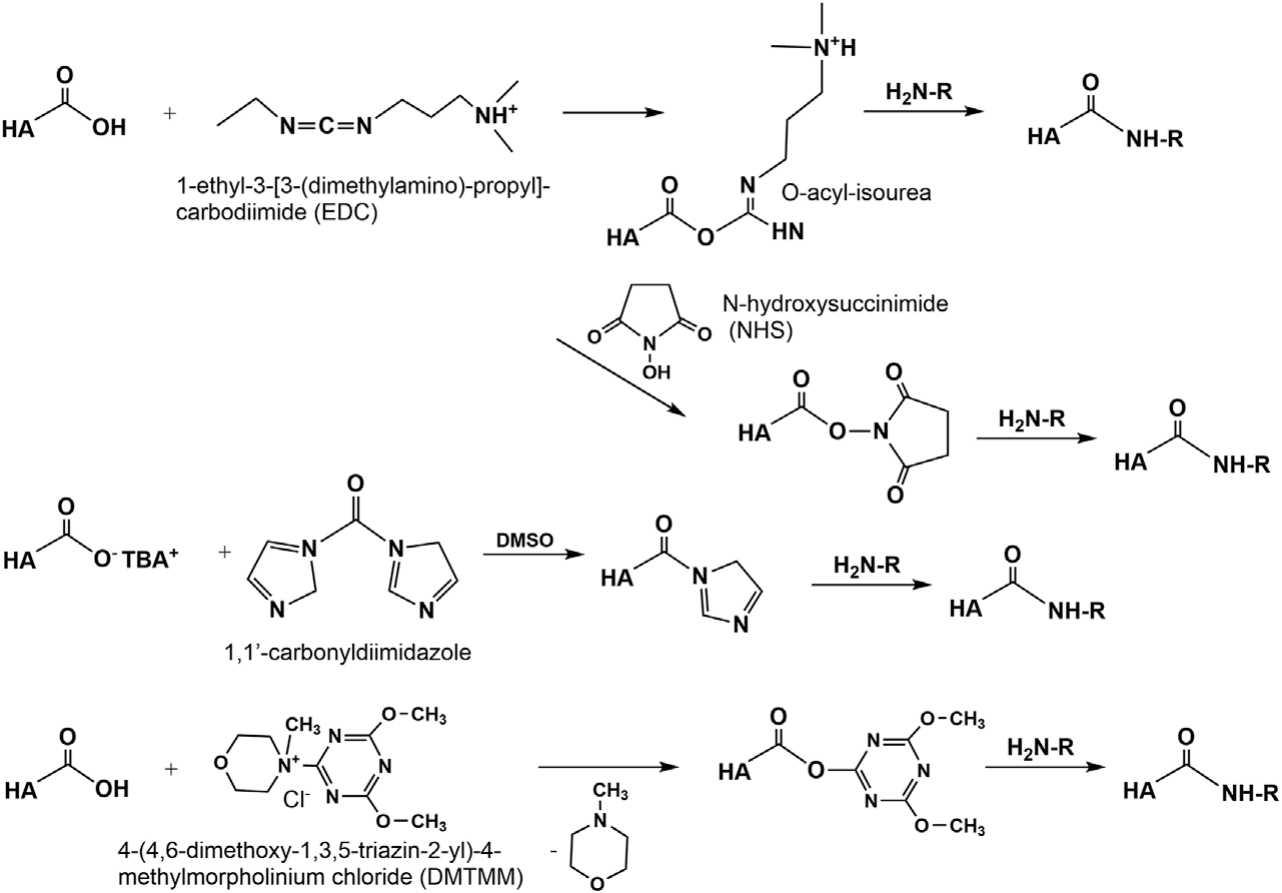
Activation of the HA carboxyl group to perform HA amidation.

A versatile chemical methodology that allows attachment of therapeutic drugs, reporter groups, crosslinking units, and other bioactive moieties to HA was developed in the Prestwich lab. Several mono-, bis-, and polyhydrazides have been attached at pH 4.0–4.75 in the presence of a water-soluble carbodiimide (EDC) to HA (Prestwich et al., 1998). Under similar conditions dithio-containing hydrazides like 3,3′-dithiobis (propanoic hydrazide) (DTP) were attached to the carboxylic HA group (Shu et al., 2002, Figure 10). In the subsequent reaction step, the corresponding HA thiols are formed by addition of DTT. This disulfide cross-linked HA hydrogel can be prepared under physiological conditions without the addition of cross-linking agents and without the production of byproducts.

**FIGURE 10:**
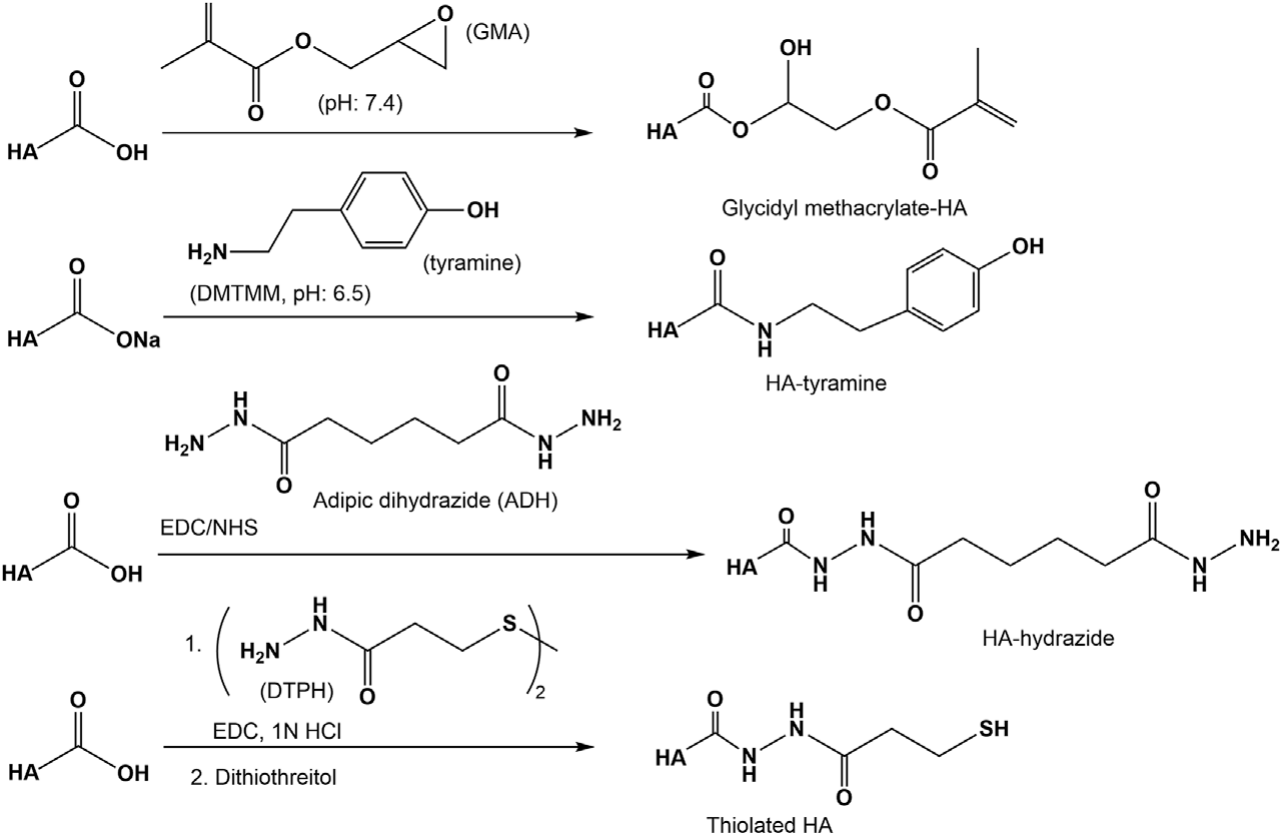
Reactions of the HA carboxyl group with epoxides, amines, and hydrazides.

Another approach to attach thiol groups to HA consist in the reaction of the HA carboxyl group with cystamine in the presence of EDC/NHS at pH 4-5 followed by cleavage of the disulfide using DTT (Jin et al., 2010, Figure 10). Using this method, also cysteine was attached via an amide linkage to the HA carboxyl group (Kafedjiiski et al., 2007). As recently shown, such thiolated HA derivatives are known for their mucoadhesive properties as well as enzyme inhibitory, permeation enhancing effects in drug delivery systems (Griesser et al., 2018).

Recently, a disulfide-based protecting group strategy for *in situ* formation of stable HA hydrogels has been developed (Ossipov et al., 2010). In this work a central divalent protecting group, 2,2′-dithiobis (ethoxycarbonyl), which links two identical molecules, was used. The designed reagents allow mild and highly controlled functionalization of HA with various types of nucleophilic chemoselective groups (Ossipov et al., 2010). The used strategy has been applied in later studies for the preparation of *in situ* forming HA gels hybridized with nanoparticles (Kheirabadi et al., 2015) or interpenetrating double HA-fibrin networks as an injectable and biodegradable scaffold for cell proliferation and differentiation (Zhang et al., 2016).

### Modification of the N-Acetyl Group of HA

Deacetylation recovering the free amino group is the main reaction type performed at the N-acetyl group of HA (Sedláček et al., 2020). Usually, deacetylation of HA occurs with hydrazine sulfate at increased temperatures over several days (Dahl et al., 1988; Crescenzi et al., 2002b). Recently, an advanced procedure, using a hydrazine/hydrazine sulfate mixture for deacetylation followed by the addition of iodic acid was described (Zhang et al., 2013; Babasola et al., 2014, Figure 11). In addition, partial deacetylation is also possible by treatment of HA with NaOH (Wada et al., 1994). In all these cases a substantial molecular weight decrease occurs. In contrast to chemical methods of N-deacetylation, enzymatic processes of N-deacetylation (Chen et al., 2009; Kim and Kim, 2011) occur at mild conditions without massive molecular weight changes (Sedláček et al., 2020). However, this process requires enzymes (HA-N-deacetylases) which are at present not well described and commercially not available. Future progress in enzyme technology will help to prepare deacetylated HA without using hazardous and HA degrading chemicals. Several subsequent reactions of the amino group of deacetylated HA including re-acylation (Babasola et al., 2014) or Ugi-reaction to form novel HA derivatives and hydrogels (Crescenzi et al., 2002b) have been described.

**FIGURE 11:**
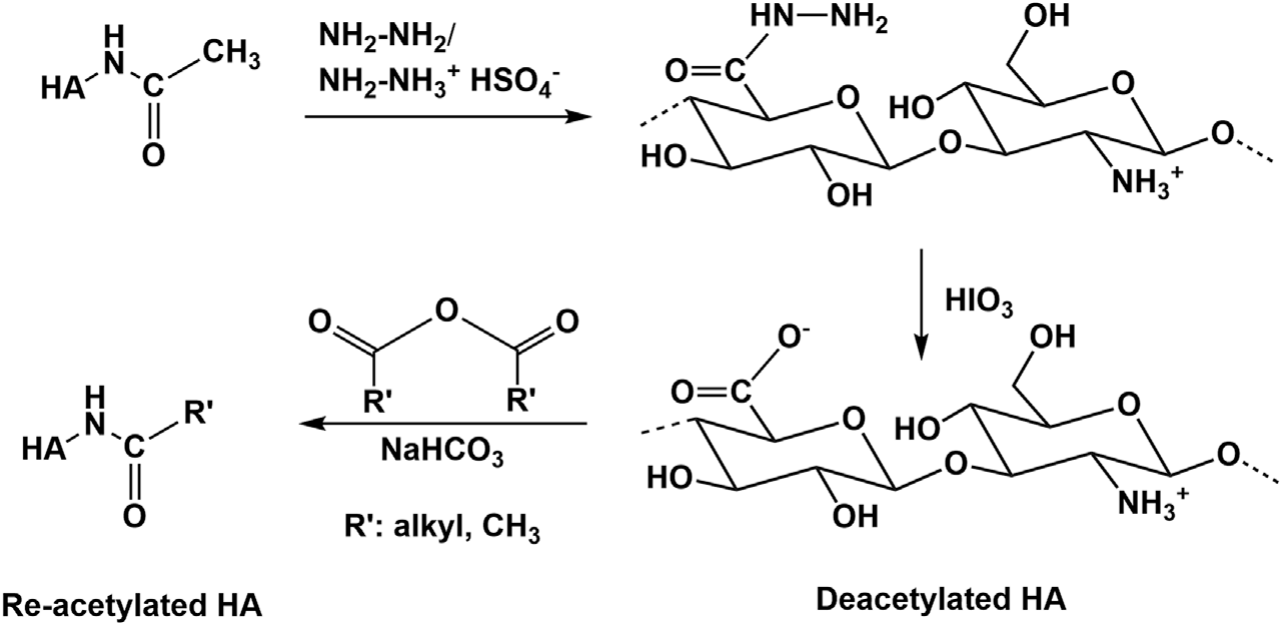
Deacetylation of HA with hydrazine.

### Conjugation of HA by Reducing End Group Functionalization

**FIGURE 12:**
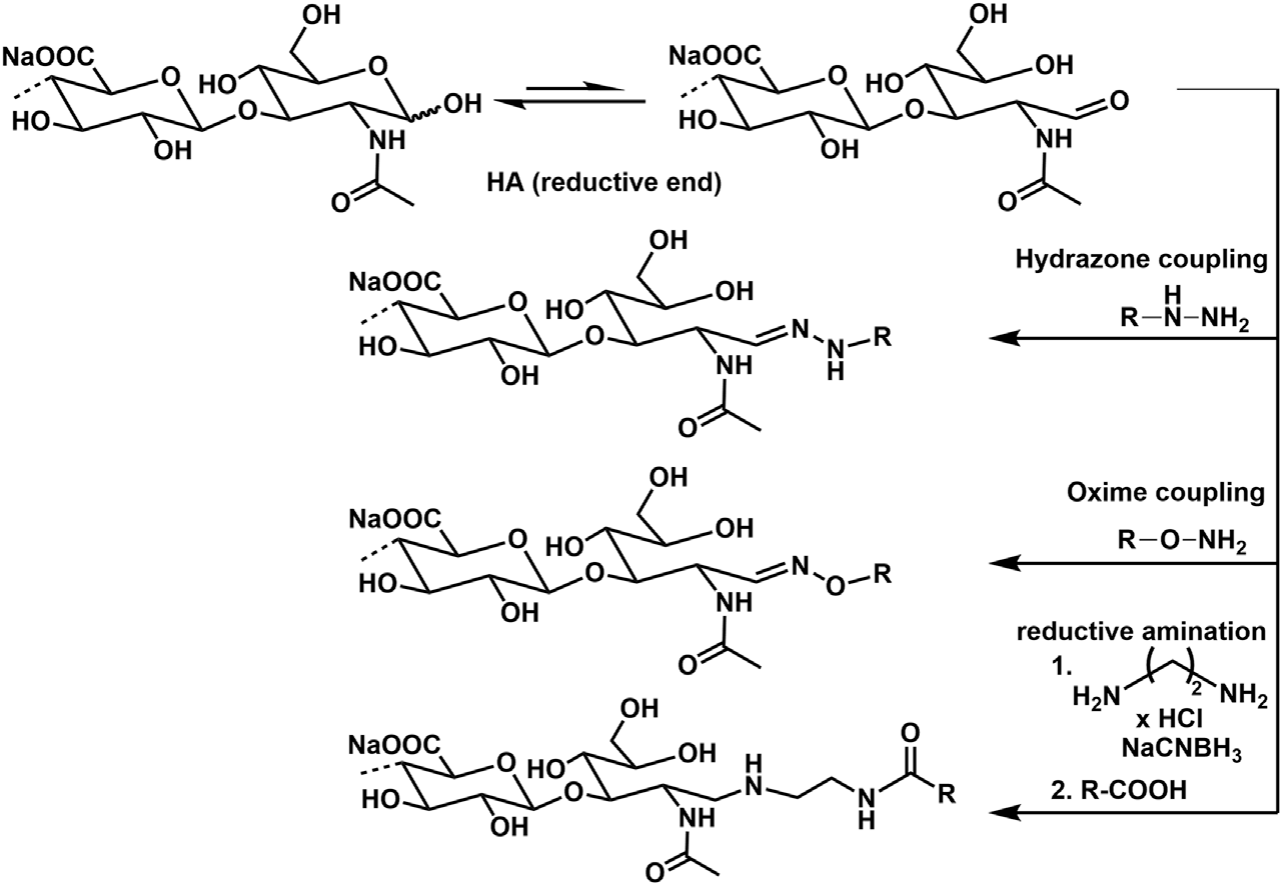
Conjugation of amino group containing molecules at the reducing end of HA.

Advanced screening applications e.g., as microarrays, functional molecular and cellular assays, as well as biosensors require the attachment of HA to surfaces or other biomaterial scaffolds. In this regard, specific conjugation of active molecules like markers through the reducing end is desirable, as it effectively mimics the cell surface presentation of HA motifs and avoids alteration of HA–peptide interactions by chemical modifications along the biopolymer chain, or by surface-imposed conformational or spatial constraints. The most frequently used techniques for this type of GAG functionalization are currently hydrazone (Altgärde et al., 2013; Peerboom et al., 2017) and oxime (Novoa-Carballal and Müller, 2012; Thakar et al., 2014) ligation chemistry. Another procedure uses reductive amination with e.g., ethylendiamine dihydrochloride and sodium cyanoborohydride (Clauder et al., 2020). Examples of conjugation reactions are presented in Figure 12.

HA functionalization at the reducing end can also be performed with a thiol group, enabling direct immobilization on gold and other metal surfaces and coupling to marker molecules as biotin. The end group functionalization is performed treating HA with cysteamine and using sodium cyanoborohydride as reductive agent (Minsky et al., 2016).

### Interaction of Chemically Modified Hyaluronan With Biological Mediator Proteins

The interaction analyses of sulfated and carboxymethylated GAG derivatives with the biological mediator proteins transforming growth factor-β1 (TGF-β1), bone morphogenetic protein-2 and -4 (BMP-2 and -4) and osteoprotegerin (OPG) were previously reviewed (Schnabelrauch et al., 2013; Scharnweber et al., 2015) and are briefly summarized here. For these interactions, sulfation degree and patterns as well as the structure of the sugar backbone were identified as major determinants for binding strength as revealed by combining immunobiochemical methods and surface plasmon resonance (SPR) with molecular modelling (Hintze et al., 2009; Hintze et al., 2012; Salbach-Hirsch et al., 2013; Hintze et al., 2014). Using the complementary experimental methods, ELISA and SPR, with either the GAG or the proteins immobilized it could be shown that the derived GAG binding profiles match in general, excluding effects due to immobilization of one interaction partner. HA derivatives were found to interact stronger than the corresponding CS derivatives with the same sulfation degree (Hintze et al., 2009; Hintze et al., 2012; Hintze et al., 2014). Since the natural CS used for sulfation contained 70% chondroitin-4-sulfate and 30% chondroitin-6-sulfate, differences in the sulfation degree of the C-4 position, next to different geometries of the sugar backbone, were identified as possible reasons for this effect. Further, the additional carboxymethylation of the high-sulfated sHA3 was detrimental for the binding strength towards TGF-β1, which was attributed to possible differences in the C-4 and C-6 sulfation due to carboxymethylation preceding the sulfation reaction (Hintze et al., 2012).

In case of BMP-2 and OPG the biological relevance of the GAG/mediator protein interaction was further investigated by including cell receptors or other natural interaction partners in the SPR interaction studies, i.e. BMP-2 receptor-IA/Fc chimera or receptor activator NF-κB ligand (RANKL) (Salbach-Hirsch et al., 2013; Hintze et al., 2014). Here, the latter were immobilized on the sensor surface and studied for their interaction with the mediator proteins pre-incubated with HA derivatives. In both cases, this resulted in a concentration- and sulfation-dependent decrease of the binding response indicating that HA derivatives interfere with the complexation of these natural interaction partners and thus reduce the biological activity of the respective biological mediators. For OPG, SPR findings on the biological relevance were supported in a RANKL-induced *in vitro* osteoclastogenesis model demonstrating a rescued osteoclast formation after pre-incubation of OPG with sulfated GAG (Salbach-Hirsch et al., 2013). In both cases, the integration of computational methods revealed how sHA derivatives influence the interplay of these mediators with their natural interaction partners up to the atomic level.

This approach was subsequently applied on further biological systems to gain a broader understanding of the structure-function relationship of GAG in their interaction with mediator proteins relevant to the healing process in bone and skin and the biological consequences of these interactions.

The biological relevance of the GAG/TGF-β1 interaction was further investigated by including its natural receptors TGF-β receptor-I (TβR-I) and -II (TβR-II) (Koehler et al., 2017). As for BMP-2 and OPG, sHA blocked binding of TGF-β1 to its receptors in a concentration- and sulfation-dependent manner, supporting a previous publication showing an impaired TGF-β1 driven differentiation of dermal fibroblasts due to TGF-β1 interaction with sHA derivatives (van der Smissen et al., 2013).

The capability of GAG derivatives to modulate canonical Wnt signaling, in particular the activity of the extracellular inhibitors sclerostin (SOST) and Dickkopf (Dkk-1) that promote osteoclastogenesis and bone resorption, was evaluated utilizing a combination of the abovementioned complementary methods (Salbach-Hirsch et al., 2015; Gronbach et al., 2020). SPR revealed that GAG interact with SOST in a concentration- and sulfation-dependent manner, with sHA3 being the strongest binder, while high-sulfated CS (sCS3) and heparin demonstrated significantly weaker binding (Salbach-Hirsch et al., 2015). In contrast, HA binding was found to be marginal. The biological relevance was verified in an LRP5/SOST interaction study and in an *in vitro* model of Wnt activation showing a sulfation- and concentration-dependent reduction in bioactivity due to the SOST/GAG interaction via interference with sclerostin/LRP5/6 complex formation.

The sulfation-dependent interaction of sHA3 with Dkk-1 was demonstrated by SPR analysis (Gronbach et al., 2020). Further, binding affinity was found to be comparable for those derivatives containing an ATTO-565 fluorescent label to non-labeled sHA3, while non-sulfated ATTO-labeled GAG showed negligible binding responses towards Dkk-1. This result was in line with findings of the same study on a macromer-based film covalently decorated with ATTO-labeled sHA3 efficiently scavenging Dkk-1 and displaying pro-osteogenic effects with SaOS-2 cells and primary human mesenchymal stem cells (hMSC).

The interaction of polymeric and oligomeric GAG derivatives with heparin-binding EGF-like growth factor (HB-EGF), a major factor activating keratinocytes and dermal fibroblasts in skin wound healing, was analyzed via SPR and molecular modeling (Thönes et al., 2019). While there was no binding detected for non-sulfated HA, CS and sHA1 were found to interact with HB-EGF. Interestingly, heparin displayed the same binding strength as sHA1 albeit a higher sulfation degree. This indicated that next to sulfation degree, the sulfation pattern and/or the structure of the sugar-backbone might play a role. This was supported by the interaction profiles of HA oligosaccharides with immobilized HB-EGF demonstrating an interaction for those molecules with at least one sulfate group at the C4 or C6 position of the N-acetylglucosamine unit. In this case, the binding strength of sHA tetrasaccharides increased with the degree of sulfation. Importantly, the interaction strength of a persulfated hexasaccharide was found to be lower than for its tetrasaccharide counterpart, highlighting an additional effect of chain length. These findings provided a rational for the development of sHA1-containing HA/collagen hydrogels that were found to improve wound healing processes *in vitro* and in a porcine skin organ culture model by the sustained release of biologically active HB-EGF.

**FIGURE 13:**
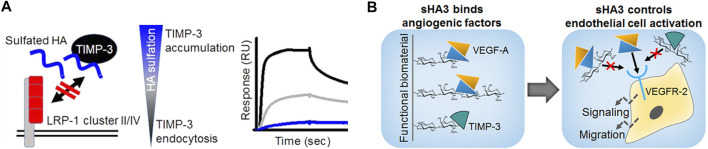
Mechanisms of GAG action on TIMP-3. **(A)** GAG control ECM remodelling via stabilizing and accruing TIMP-3, interfering with TIMP/LRP-1 complex formation and increasing extracellular TIMP-3 level; SPR sensorgram: binding analysis of solute TIMP-3 with immobilized LRP-1 cluster II in the absence or presence of differently sulfated sHA (black line: interaction of TIMP-3 alone, grey and blue line: interaction with TIMP-3 after preincubation with sHA1 and sHA3, respectively). **(B)** GAG control endothelial cell activation by interfering with VEGFR-2 complexation via VEGF-A and TIMP- (Reprinted with permission from Rother S. et al. 2016 Biomacromolecules 17, 3252-3261 and Rother S. et al. 2017 ACS Appl. Mater. Interfaces 9, 9539-9550. Copyright ũ 2016/2017 American Chemical Society).

A particular striking example of how biophysical/biochemical interaction analyses in combination with computational methods and *in vitro* cell culture models move forward insights into the mechanism of GAG action, are a compendium of studies regarding the tissue inhibitor of metalloproteinases-3 (TIMP-3). First of all, the molecular interplay of polymeric and oligomeric GAG derivatives with TIMP-3 was demonstrated by SPR, molecular modeling and hydrogen/deuterium exchange mass spectrometry (Rother et al., 2016a). Interestingly, unlike for previously investigated mediators, the interaction of sHA derivatives was found to be of comparable strength than for the corresponding sCS derivatives with the same sulfation degree. Importantly, this interaction did not limit the capacity of TIMP-3 to inhibit matrix metalloproteinase-1/-2 (MMP-1/-2) in enzyme kinetics, since no overlap of the binding sites was revealed. These observations pointed towards a novel strategy for controlling ECM remodelling by GAG, e.g. in chronic wound situations, via stabilizing and accruing TIMP-3, while maintaining its inhibitory activity towards MMP activity. This hypothesis was further corroborated by the observation that TIMP-3 directly interacts with clusters of the endocytic receptor low-density lipoprotein receptor-related protein (LRP-1) (Rother et al., 2016b; Figure 13A). GAG were shown to interfere with this TIMP-3/LRP-1 complex formation in a sulfation-dependent manner. Further, sHA1 was found to increase the extracellular TIMP-3 level of hMSC. Thus, sHA-containing biomaterials might be promising to interfere with pathological matrix degradation and thereby encourage wound healing. Finally, a novel mechanism was identified by which GAG might control angiogenic processes (Rother et al., 2017b; Figure 13B), which could be of interest in angiogenesis-related diseases. By blocking the binding of both, VEGF-A and the angiogenic inhibitor TIMP-3, to the VEGF-A receptor VEGFR-2, GAG reduce the biological activity in a sulfation-dependent manner. When TIMP-3/sHA complexes were simultaneously forming, VEGF-A/VEGFR-2 signaling was partially rescued. While labeling sHA3 with an ATTO-565 fluorescent label did not change the binding characteristics of sHA3 with immobilized Dkk-1 as mentioned above (Gronbach et al., 2020), acrylation significantly reduced binding strength of the low sulfated HA derivatives, sHA1 and sHA1Δ6s, towards immobilized VEGF-A in SPR analysis as well as in molecular docking and dynamics (MD) simulations (Rother et al., 2021). This was supported by a solid-phase binding assays with immobilized GAG demonstrating a significantly negative effect of acrylation for sHA1Δ6s suggesting that the presence of acrylate groups alter the accessibility of the sulfate groups and/or have a detrimental effect on the optimal carbohydrate conformation. When translated to sHA-containing acrylated HA/collagen hydrogels, this led to biomaterials releasing biologically active VEGF-A in a defined manner depending on the substitution pattern of the sulfated GAG. In turn angiogenic processes like endothelial cell proliferation and the formation of an extended morphology, indicating sprouting, could be controlled. Together with the findings of Thönes et al., it can be concluded that multivalent carbohydrate-based hydrogels containing acrylated sHA derivatives are promising for reaching distinct growth factor delivery profiles, increasing the healing capacity of vascularized tissues.

## HA as Biomedical Polymer Matrix

Native HA is biofunctional and forms viscoelastic biocompatible and degradable polymer networks making it an interesting material for medical and pharmacological applications. However, modifying HA is especially favorable to overcome certain limitations of native HA such as the quick enzymatic degradation and rapid dissolution in water (Smejkalová et al., 2012b). A higher enzymatic resistance as well as tunable solvation profiles are often prerequisites for biomedical applications. The simplest usage of HA is in form of solutions in physiological buffers, where different properties are achieved by varying the concentration as well as the molecular weight of HA (Nicholls et al., 2018). Hybrid complexes composed of thermally treated HMW-HA and LMW-HA based on biophysical interactions between hydrogen bonds of HA chains are explored as injectable polymer matrix especially for the treatment of osteoarthritis (Stellavato et al., 2019; La Gatta et al., 2021).

**FIGURE 14:**
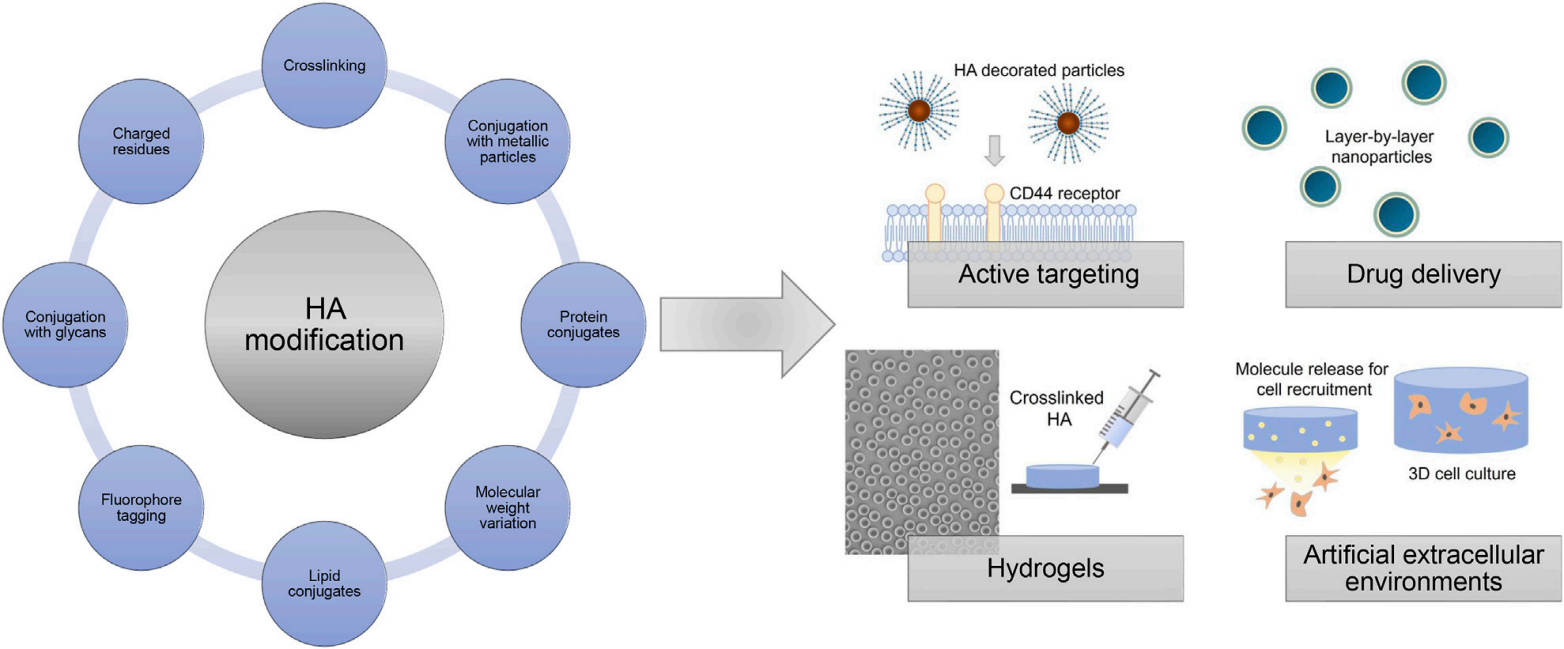
Overview of HA modification strategies and selected applications.

Chemical modification strategies targeting the preponderantly present carboxylic acid groups, or primary and secondary hydroxyl groups and, after deamidation, the *N*-acetyl groups, give rise to HA derivatives with altered biological as well as material properties (Figure 14). The introduction of hydrophobic groups allows to encapsulate for example, hydrophobic drugs in amphiphilic HA, which was used for active targeting and drug delivery (Smejkalová et al., 2012b; Huerta-Angeles et al., 2014; Kwag et al., 2014; Huerta-Angeles et al., 2016). Achieving tailored HA properties depends on precise and effective synthesis strategies. In this regard, functionalized HA can be used to generate films and hydrogels after crosslinking with different mechanical properties as sprays, wound dressings, scaffolds for tissue engineering approaches or as anti-adhesive materials (Eng et al., 2010; Gebe et al., 2017; reviewed in Burdick and Prestwich, 2011).

In addition, HA derivatives conjugated with metallic particles, like gold nano particles, are explored for targeted treatment of infected and cancer cells (Lee et al., 2012; Sanfilippo et al., 2020). HA-protein conjugates using for example, growth factors have been produced to mask the protein activity and to extend the protein stability (Ferguson et al., 2010; Altiok et al., 2016). Commercially available degradation assays use immobilized biotin-tagged HA as substrate to determine hyaluronidase activities (Krupkova et al., 2020). Another way to use HA for mimicking the native cell microenvironment is by physically entrapping HA of different molecular weights within a polymer network like collagen fibers (Rother et al., 2015; Unnikandam Veettil et al., 2021).

The negative charge of HA allows for non-specific adsorption on cationic surfaces such as poly (l-lysine)-coated substrates and layer-by-layer approaches (Dreaden et al., 2014; Kocourková et al., 2021). Bisphosphonate-functionalized HA was used as injectable non-covalently self-assembling hydrogel with reversible calcium binding properties (Nejadnik et al., 2014). Surfaces coated with HA-binding proteins like aggrecan globular domain 1 (HA binding protein, HABP) or HA binding peptides allow for a more selective capturing of HA, which can be used for potential interaction studies e.g. in form of enzyme-linked immunosorbent assays and GAG-protein microarrays (Yuan et al., 2013; Faust et al., 2018). HA derivatives with introduced negatively charged residues like sulfate groups and networks composed of HA covalently linked to sulfated GAG derivatives like chondroitin sulfate are also exploited as platforms with tunable growth factor presentation and release to direct cellular behavior (Ni et al., 2015; Thönes et al., 2019). Furthermore, HA derivatives with covalently attached fluorophores are widely applied as tools studying the potential association and translocation of HA in biological contexts (AlKhoury et al., 2020). Overall, modified HA derivatives are widely used and explored in different biomedical fields and applications repeatedly indicating the great promise for future customized therapies.

Due to its transparency and its lubricating and hydrophilic properties, HA solutions are often used during ophthalmic surgery and for ophthalmic preparations such as eye drops treating the dry eye syndrome (Huerta Angeles and Nesporova, 2021). Furthermore, HA is widely applied as part of contact lenses, for surface modifications of medical lenses and for sustained drug release to treat eye-related diseases (Chang et al., 2021). Subcutaneous injections of HA solutions and *in situ* forming hydrogels are applied in aesthetic dermatology for reducing wrinkles, folds, and augmenting for example, lips (Rah, 2011). In addition, viscosupplementation with HA formulations is often used to treat osteoarthritis in the knee and in other joints by reducing the mechanical stress on the joints (Henrotin et al., 2015).

## Perspectives and Concluding Remarks

In the last decades, the development of HA as a biomaterial has been a major success due to the good industrial availability of pure HA and its properties including an excellent biocompatibility, an adjustable biodegradability, mucoadhesiveness and viscoelasticity.

This review aims to provide a short overview about known and suitable chemical methods to modify the natural GAG, HA, which is of particular interest in the field of tissue regeneration and regenerative medicine. Due to the mentioned challenges of limited HA solubility in organic solvents and its sensitivity to more drastic chemical reaction conditions with regard to oxidative or thermal stress, and both strong acidic or alkaline environment, the controlled chemical modification of this multifunctional polysaccharide is still a challenging task. Nevertheless, during the last decade, numerous regioselective syntheses routes could be elaborated, which proceed with high control of regioselectivity regarding the degree and the position of substitution along the HA chain as well as with largely preservation of the HA molecular weight. At last, this could also be achieved by the advancement of modern polymer analytics, especially high-resolution NMR-spectroscopy. A current trend is the combination of conjugation and cross-linking processes using bioactive molecules for applications in regenerative medicine, pharmaceuticals and bioengineering, including cell and bioactive molecule delivery combined with tissue engineering scaffolds (Knopf-Marques et al., 2016; Fallacara et al., 2017). Although HA displays a great number of potential applications, further studies and technological advancement are necessary as there are still some open questions to be answered. The key mechanisms that control the molecular weight of HA during biotechnological synthesis has to be clarified to develop methods for the production of defined HA with uniform size (Fallacara et al., 2018). Also the reproducibility of the preparation of HA derivatives during scale-up processes has to be improved as a basis for their successful commercialization.

A wide range of biological activities has been investigated using modified HA derivatives and some of these applications are highlighted in this paper. HA has a broad scientific capability and especially the role of sulfated HA in viral infections, and the potential of HA and other GAG to address numerous medical and biotechnological challenges is becoming without any doubt the focus of renewed attention.
